# Genetic basis of dental agenesis - molecular genetics patterning clinical dentistry

**DOI:** 10.4317/medoral.19158

**Published:** 2013-10-13

**Authors:** Nidhi Chhabra, Mridula Goswami, Anuj Chhabra

**Affiliations:** 1 Department of Pedodontics & Preventive Dentistry, Maulana Azad Institute of Dental Sciences, Delhi, India; 2 Department of Dental Surgery, Safdarjang Hospital, Delhi, India

## Abstract

Tooth agenesis is one of the most common congenital malformations in humans. Hypodontia can either occur as an isolated condition (non-syndromic hypodontia) or can be associated with a syndrome (syndromic hypodontia), highlighting the heterogeneity of the condition. Though much progress has been made to identify the developmental basis of tooth formation, knowledge of the etiological basis of inherited tooth loss is still lacking. To date, the mutation spectra of non-syndromic form of familial and sporadic tooth agenesis in humans have revealed defects in various such genes that encode transcription factors, MSX1 and PAX9 or genes that code for a protein involved in canonical Wnt signaling (AXIN2), and a transmembrane receptor of fibroblast growth factors (FGFR1). The aim of this paper is to review the current literature on the molecular mechanisms responsible for selective hypodontia in humans and to present a detailed overview of causative genes and syndromes associated with hypodontia.

** Key words:**Tooth agenesis, hypodontia, growth factors, mutations.

## Introduction

Hypodontia (dental agenesis) is the most common developmental anomaly in humans, constituting a clinically challenging problem. Hypodontia is often used as a collective term for congenitally missing teeth, although specifically, it describes the absence of one to six teeth, excluding third molars. Oligodontia (multiple aplasia) refers to the congenital absence of six or more teeth, excluding third molars. Anodontia represents a complete failure of one or both dentitions to develop (1). Hypodontia is usually associated with other oral anomalies, such as cleft lip and/or palate, reduction in size and form of teeth and alveolar processes, short root anomaly, crowding and/or malposition of other teeth, delayed formation and/or delayed eruption of other teeth, persistent deciduous teeth, impaction, anomalies of the enamel, increased freeway space, false diastema, deep overbite, taurodontism, maxillary canine/first premolar transposition, enamel hypoplasia, and altered craniofacial growth (2-5).

Hypodontia can either occur in association with other genetic diseases as part of a recognized clinical syndrome, or as a non-syndromic, familial form, which occurs as an isolated trait, shows a wide phenotypic heterogeneity, appears either sporadically or in a familial fashion within a family pedigree (6). Although dental agenesis is occasionally caused by environmental factors, such as infection (e.g. rubella), various kinds of trauma of the dental region, multi-reagent chemotherapy or radiotherapy, or disturbances in jaw innervations, in a majority of cases, hypodontia has genetic causes (1,6). While a number of clinical studies have been carried out on disorders that involve the congenital lack of teeth, until recently, a very little effort has been made to understand the genetic module accountable for mammalian tooth development. Advancements in molecular biology approaches coupled with the now complete human genome sequence has allowed a number of putative disease genes/loci associated with the hypodontia/oligodontia phenotypes to be identified (7).

The knowledge of the genotype–phenotype correlation between mutations and teeth agenesis is important for genetic counseling, for a more comprehensive evaluation of the patient, and for anticipating suitable management of the dental abnormalities, especially in children.

## Molecular Mechanisms Involved in Odontogenesis

Recently, a number of genes have been identified that are involved in tooth morphogenesis and their regulatory role throughout the development of the tooth organ, i.e. from tooth initiation to tooth patterning (determination of the location, identity, size and shape of teeth) and histogenesis of the dental tissues has been highlighted (8). Previous studies showed that some genes have a strong influence on tooth development (MSX1, PAX9, LEF1, PITX2), whereas other genes have a less pronounced effect (DLX1, DLX2, GLI2, GLI3) (8-10). PAX9 has been identified as a key controlling factor during the odontogenic process with its expression found specifically at the prospective sites of all teeth prior to there being any morphological signs of odontogenesis (7). A general role for MSX1 in the development of ectodermal derivatives has been suggested with it strongly expressed in the dental mesenchyme (7).

In mammals, tooth development is governed by a sequential and reciprocal signaling process between two adjacent tissues, the primitive epithelium lining the stomodeum and mesenchymal cells arising from cranial neural crest cells (11) ( Table 1). The oral epithelium initiates tooth development at embryonic day 9-11 by signaling through generic molecules such as Fgfs, Bmps, Wnt, and Shh and continue to be involved in further morphogenesis and cytodifferentiation of the tooth (12). Signaling molecules that determine the position and the shape of the teeth are MSX1, MSX2, DLX1, DLX2, BARX1, and PAX (13). PAX9 and MSX1 have been reported to have an important regulatory role in the maintenance of BMP4 expression and signaling, implying they may also have a role in odontogenic potential shifts (13).

**Table 1:**
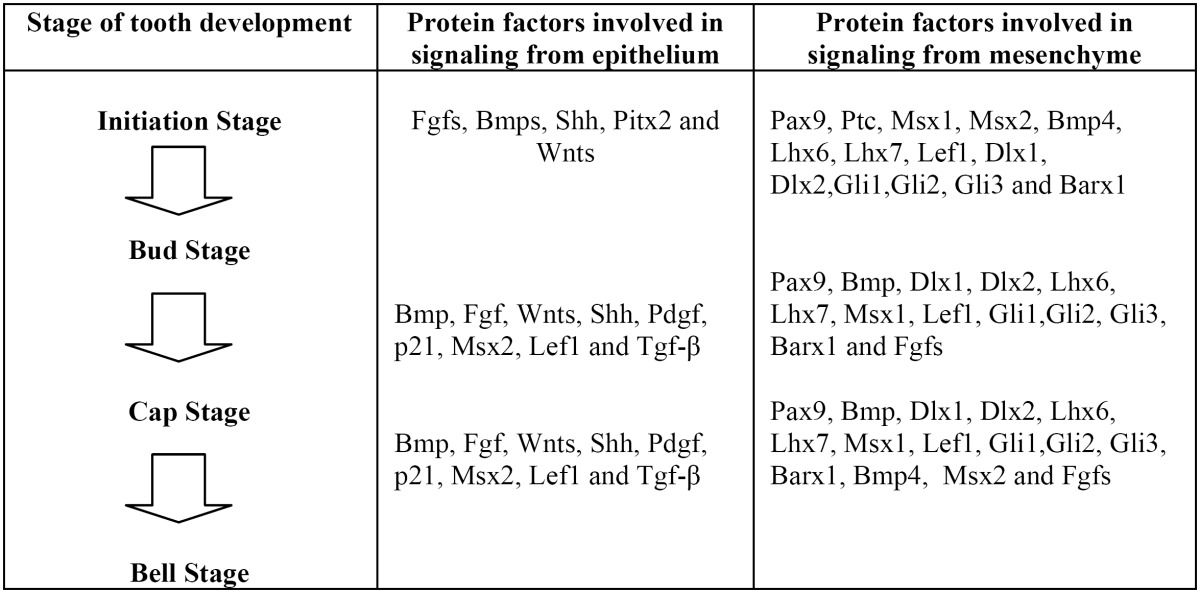
Signaling protein factors involved in tooth development, failure of one of which may result in patterning defects.

A variety of dental anomalies either morphological, numerical, and/or structural in nature may result due to abnormal function of these specific proteins. Depending on the molecule and its timing of required expression in either (or both) the oral epithelium and adjacent mesenchyme, tooth primordia may be absent (Wnt, p63), or tooth development may be arrested at the bud stage (Lef1, Msx1, Msx2, Pax9, Pitx2) or at the cap/bell stage (Cbfa1/Runx2) (14-19). Studies have shown that tooth development is arrested at the bud stage in both Pax9 and Msx1 mutant mice (17), suggesting they have similar, non-redundant roles in signal progression to the cap stage of tooth development.

## Genetics of Human Tooth Agenesis

Modern molecular genetic techniques have helped us to identify the genetic factors responsible for tooth agenesis and the mechanisms responsible for tooth agenesis but more studies are required to discover how malfunctions in these factors disrupt tooth development. Using gene mapping techniques on families known to have hypodontia and/or oligodontia investigators have been able to definitively link several gene mutations with tooth agenesis.

## Non-Syndromic Hypodontia

Non-syndromic hypodontia is by far the most common form of congenital tooth absence and can involve variable numbers of teeth. It is more commonly seen in the secondary dentition and is rare in primary dentition. Non-syndromic hypodontia is classified as a sporadic or familial form, inherited in an autosomal-dominant, autosomal-recessive or X-linked mode, with considerable variation in both penetrance and expressivity (20). To date, the mutation spectra of non-syndromic form of familial and sporadic tooth agenesis in humans have revealed defects in various such genes that encode transcription factors, MSX1 and PAX9 or genes that code for a protein involved in canonical Wnt signaling (AXIN2), and a transmembrane receptor of fibroblast growth factors (FGFR1) ( Table 2). Protein products of genes that encode transcription factors - MSX1 and PAX9, are responsible for the crosstalk between dental tissues and are essential for the establishment of the odontogenic potential of the mesenchyme (12).

**Table 2:**
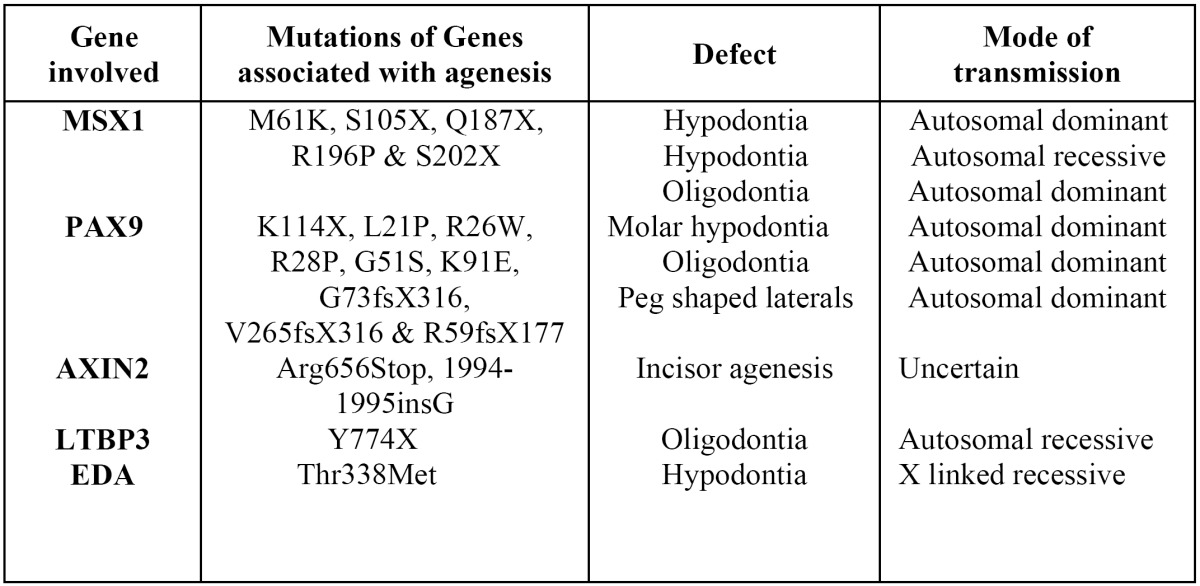
Genes associated with tooth agenesis in humans.

MSX1

MSX1 contains a highly conserved homeobox sequence encoding a 60 amino acid-long DNA-binding homeodomain (21). MSX1 belongs to a family of transcription factors that are expressed in overlapping patterns at multiple sites of tissue interactions during vertebrate development (22). Till date, five point mutations have been identified within MSX1 gene mutations, with two leading to a substitution mutation within the protein and the remaining three forming a stop codon that prematurely truncates the protein. Two mutations fall within the N-terminal region prior to the central homeodomain (M61K & S105X), with the remaining three (Q187X, R196P & S202X) all falling within the homeodomain itself.

Of the two substitution mutations, the M61K mutation falls outside of the homeodomain of MSX1, and it has been reported that it may cause disruption of protein interactions. The R196P mutation falls within helix-I of the MSX1 homeodomain, disrupting its stability and functional activity (23). Of the three premature termination mutations, S105X is the only mutation to occur prior to the homeodomain of MSX1. The remaining two termination mutations fall within the central region of the MSX1 homeodomain. However, there is no clear correlation between the severity of the hypodontia and the severity of the effect on the MSX1 protein caused by the identified missense mutations.

PAX9

PAX9 is a member of a gene family encoding transcription factors that play a key role during embryogenesis. Proteins encoded by PAX genes share a unique 128-amino acid long DNA-binding paired domain (24). PAX9 gene products function primarily by binding the enhancer DNA sequences and by modifying transcriptional activity of downstream genes (25). To date, 11 distinct disease-causing mutations in the PAX9 gene (59 patients in 15 families) have been identified in humans, most of which are loca-ted in the paired box domain of PAX9. In contrast to MSX1, both missense and frame-shift mutations in PAX9 have been associated with hypodontia.

Of the seven identified missense mutations, one is a premature termination mutation (K114X), and the remaining six are all resi-due substitution mutations. Of these substitution mutations, only five generate a substitution in the protein (L21P, R26W, R28P, G51S & K91E), with one believed to prevent PAX9 expression. Three frame-shift mutations have been identified, two of which are caused by the insertion of a single nucleotide (G73fsX316 & V265fsX316) and the other by the deletion of eight nucleotides with the insertion of 288 foreign nucleotides (R59fsX177) (26).

The only substitution mutation to cause premature termination was an A340T switch, which creates a stop codon at lysine 114, producing a truncated PAX9 protein that terminates at the end of the N-terminal DNA binding region of the PAX9 paired-box domain (27). The remaining three missense mutations (R26W, R28P and G51S) that leads to a residue substitution in the PAX9 protein have been identified recently.

It is significant to note that most of the PAX9 frame-shift, deletion and missense termination mutations cause hypodontia in both the permanent and the primary dentitions, whereas missense substitution mutations affect the permanent dentition only.

MSX1 and PAX9 

MSX1 and PAX9 interact during odontogenesis at both the gene and protein level and are intimately involved in the genetic networks regulating tooth development. PAX9 forms a physical association with MSX1, and this interaction takes the form of a heterodimeric protein complex, which enhances the ability of PAX9 to activate both MSX1 and mesenchymal Bmp4 gene expression during tooth development. This interaction ultimately drives morphogenesis of the dental organ, more in particular, the transition from bud to cap stage during tooth development and enamel knot induction at the late cap stage (28).

Besides Bmp4 downregulation, mutations in PAX9 could result in a selective reduction in PAX9 binding to sites that regulate MSX1 expression levels. Mutations in either PAX9 or MSX1 can also lead to defective protein–protein interactions, both at the gene and protein levels that disrupt normal downstream functions important for tooth morphogenesis (28).

Haploinsufficiency of MSX1 protein affects the development of all teeth, specifically third molars and second premolars, while reduced amount of PAX9 protein mainly affects molar development.

AXIN2

AXIN2 or axis inhibitor protein 2 is a gene located on the long arm of chromosome 17 with a genetic address of 17q23-q24. The association of the gene to tooth agenesis was first found in a Finnish family with a predisposition for colorectal cancer (29). The mutations of AXIN2 -Arg656Stop and 1994-1995insG lead to decreased AXIN2 function and most probably represent loss-of-function mutations that cause activation of Wnt signaling. AXIN2 was selected as a strong candidate gene for several reasons: Its position within this particular chromosomal region, a previously identified association with colorectal carcinoma and the fact that AXIN2 is also a known regulator of the Wnt signalling pathway. The Wnt family of secreted proteins forms part of a large family of signalling molecules that have a wide-ranging role during embryonic development and demonstrate regionally restricted expression in the tooth (30).

The mode of transmission of hypodontia due to defects in the AXIN2 gene has not been definitively proven, and it has been seen that individuals with a non-sense mutation in AXIN2 display a mixed pattern of dental agenesis.

LTBP3

LTBP3 (latent transforming growth factor beta binding protein 3) is a gene that modulates the bioavailability of TGF-beta and is located on the long arm of chromosome 11. A study on a Pakistani family with a history of consanguineous marriage found that a mutation in the LTBP3 gene causes an autosomal recessive form of familial oligodontia (31).

EDA 

EDA (ectodysplasin 1) is a gene located at Xq12-q13.1 that has been linked to X-linked recessive ectodermal dysplasia. A study of Chinese families with non syndromic X-linked hypodontia has shown that a Thr338Met mutation of the EDA gene was responsible for the congenital absence of maxillary and mandibular central incisors, lateral incisors, and canines, with the high possibility of persistence of maxillary and mandibular first permanent molars (32).

## Syndromic Conditions Associated with Dental Agenesis

Online Mendelian Inheritance in Man (OMIM) lists over 60 different syndromic conditions that include hypodontia as part of their phenotypic spectrum of anomalies and candidate genes have been identified for many of these conditions ( Table 3). Ectodermal dysplasia, oral-facial-digital syndromes, and syndromes with oral-facial clefting such as Pierre-Robin sequence and Van Der Woude syndrome are conditions, which are associated with hypodontia. Successive linkage analysis studies have indicated involvement of different loci, mapped respectively to chromosome 6p24; 2p13; 19q13; and regions on 4q, in non-syndromic cleft lip and/or palate (CL/CLP) (33). In Pierre- Robin syndrome, a 50% prevalence of hypodontia, most frequently of mandibular teeth, while in Van Der Woude syndrome (VWS), a 70% prevalence of hypodontia has been reported. Mutations in IRF6 (1q32-q41) have been identified in 50 unrelated families with VWS (34).

**Table 3:**
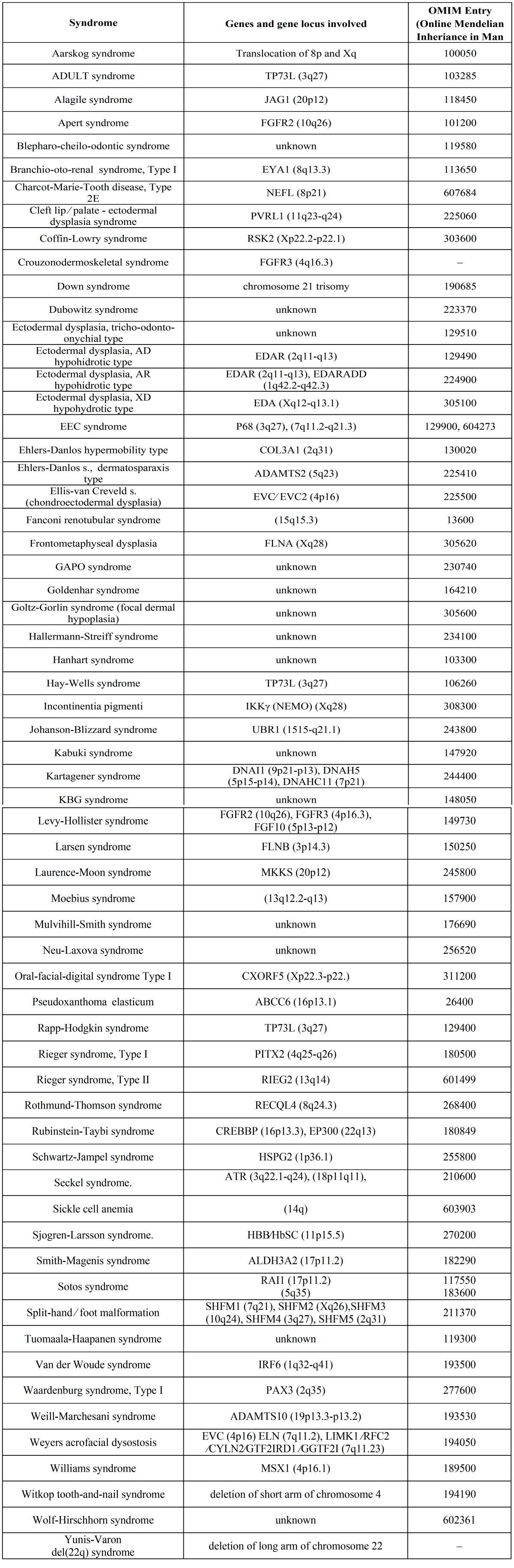
Syndromes associated with hypodontia.

Ectodermal dysplasia (ED) which is classified into 11 clinical subgroups displays a great genetic heterogeneity. X-linked dominant ED is caused by mutations in EDA gene (Xq12-q13.1). Both autosomal dominant and recessive forms of ED are caused by mutations in EDAR (2q11-q13) coding for a TNF receptor (35). Several different mutations of P68 gene (3q27), linkage to chromosome 7 (7q11.2-q21.3) and the chromosome 19 pericentromeric region have been revealed in ectrodactyly ectodermal dysplasia – orofacial cleft (EEC) syndrome families (36,37). Oral-facial-digital syndrome type I (OFD1) transmitted as an X-linked dominant condition has been associated with several mutations in CXORF5 (Xp22.3-p22.2) (38).

Mutations in MSX1 gene (on 4p16.1) have been identified in several unrelated families with Witkop tooth-and-nail syndrome. Hypodontia features in a number of other syndromes such as Rieger’s syndrome, Oculo-facial-cardio-dental syndrome, Incontinentia pigmenti, Pierre Robin sequence, Fried syndrome, Book syndrome, Down’s syndrome, Wolf-Hirschhorn syndrome, Kabuki syndrome, Diastrophic dysplasia (DTD), Hemifacial microsomia and Recessive incisor hypodontia (RIH) ( Table 3).

## Conclusions and Future Perspectives

Tooth agenesis is genetically and phenotypically a heterogeneous condition, caused by several independent defective genes, which act along or in combination with other genes and lead to specific phenotypes. During the past decades, significant efforts have been made for detecting gene loci that contributes to dental agenesis. However, there is a dearth of knowledge of the genetic epidemiology of dental agenesis and only few genotype-phenotype correlations have yet been established in humans with non-syndromic hypodontia.

Further research of genetic and pathogenetic mechanisms involved in both syndromic, and non-syndromic hypodontia is warranted to shed light into the pathogenesis of tooth agenesis, to describe the pattern of occurrence and of the malformations found on the teeth present.
